# Omics Data and Data Representations for Deep Learning-Based Predictive Modeling

**DOI:** 10.3390/ijms232012272

**Published:** 2022-10-14

**Authors:** Stefanos Tsimenidis, Eleni Vrochidou, George A. Papakostas

**Affiliations:** MLV Research Group, Department of Computer Science, International Hellenic University, 65404 Kavala, Greece

**Keywords:** artificial intelligence, deep learning, biological data, omics, drug discovery, system biology, complex systems, review

## Abstract

Medical discoveries mainly depend on the capability to process and analyze biological datasets, which inundate the scientific community and are still expanding as the cost of next-generation sequencing technologies is decreasing. Deep learning (DL) is a viable method to exploit this massive data stream since it has advanced quickly with there being successive innovations. However, an obstacle to scientific progress emerges: the difficulty of applying DL to biology, and this because both fields are evolving at a breakneck pace, thus making it hard for an individual to occupy the front lines of both of them. This paper aims to bridge the gap and help computer scientists bring their valuable expertise into the life sciences. This work provides an overview of the most common types of biological data and data representations that are used to train DL models, with additional information on the models themselves and the various tasks that are being tackled. This is the essential information a DL expert with no background in biology needs in order to participate in DL-based research projects in biomedicine, biotechnology, and drug discovery. Alternatively, this study could be also useful to researchers in biology to understand and utilize the power of DL to gain better insights into and extract important information from the omics data.

## 1. Introduction

Next-generation sequencing technologies have quickly emerged in the 21st century [1,2] and along with a number of theoretical advancements, they have resulted in a big bang-type of advance in biological knowledge. The knowledge of life is nowadays greater than it ever has been before, highlighting that living systems are immeasurably more complex than they were previously imagined to be. The volume of data that are needed to capture the biological information is vast. A human genome sequence is comprised of three billion characters (base pairs) [3], with the sequenced DNA string of a single individual taking up more than a gigabyte of memory space. The human body is estimated to contain between 80 and 400 thousand proteins [4], and the neXtProt database contains more than nine million entries of proteins and variants, which are accompanied by metadata [5]. The estimates for the number of genes in the human genome have reached up to 120 thousand [6], and gene expression datasets include thousands of features. On top of that, sequencing and other assorted technologies are continually becoming cheaper and have a higher resolution, thereby resulting in ever more data.

It has already been a decade since genomic sequencing became cheaper, and this occurred more quickly than the storage space and computing power could expand; the volume of the biological data are, therefore, rising faster than the human capacity to store and process it [7]. The information that could be extracted with the data-driven methods from such huge repositories could be significant. The relatively limited application of predictive modeling in the field of life sciences constitutes a bottleneck, thereby hampering further scientific progress. To an extent, the data and the labels are already available [8]; it remains for scientists to deploy algorithms to extract insights from these data.

When it comes to data with large volumes, dimensionality and complexity, deep learning (DL) appears to be more powerful than machine learning (ML) is. Volume and dimensionality refer to the number of rows and columns in a tabular dataset, respectively, and a high degree of data complexity means that the function mapping input and output are non-linear and have a large number of parameters. As the biological data are becoming increasingly larger in their volume, dimensionality, and complexity, DL seems to be necessary since no other method could handle efficiently the big data and the complex systems of this domain.

The introduction of DL in life sciences is already making a positive impact. In drug research, scientists often rely on quasi-random searches, arbitrarily applying chemical agents to cells and observing the effects. This immensely expensive and time-consuming approach has an estimated cost of 2.6 billion USD to develop a single drug, with 90% of the researched drugs never achieving approval or being produced [9]. The employment of computational drug discovery by using big data and deep neural networks to detect the complex patterns in drug-related databases, generate hypotheses and reduce the search space could bring a new era in pharmaceutics [10,11]. Apart from the predictive tasks, DL is also used for knowledge discovery. The learned internal parameters of a Deep Belief Network (DBN) were interrogated to infer the protein interaction networks [12], and a Deep Neural Network (DNN) that was trained on metabolic profiles was used to discover disease biomarkers [13]. The DL models have also aided scientific discovery indirectly as a part of larger workflows. U-nets played a role in the data pipelines of a project that elucidated the mechanisms and genetic pathways that are related to type-2 diabetes [14]. The Convolutional Neural Networks (CNN) detected the modifications in DNA sequences as part of a research effort to identify the genes and pathways that are correlated with aging [15].

Although applying predictive modeling in biotech is a high priority and DL seems to be the most effective method, most biologists are not familiar with DL. Attempts have been made to familiarize them with it, which has been proven by the numerous introductory surveys that have appeared in the biology-related literature [16,17,18,19,20]. However, the scope of these overviews is limited, thus pointing out that the biology-related literature is lacking in the number of DL experts. Most of the systematic reviews on the subject of DL in life sciences seem to exclusively address biologists as they assume that the reader is versed in omics, and thus, they do not provide explicit information on the data that the DL models were trained on or they assume that the reader is an absolute beginner in neural networks, thus they never go beyond the basics, avoiding sophisticated expositions of the applied architectures.

The objective of this work is to alleviate the latter two shortcomings. The motivation for this is based on realizing that the current obstacles to biomedical breakthroughs could be overcome by either teaching cutting-edge computer science to biologists or by bringing computer scientists into the field of biology. The aim of this work is to offer a survey of the various types of omics data and data representations that are used to train deep neural networks, the neural network architectures that have been implemented, and the various biological problems that DL has been solving over the last few years. The input data, the DL models, and the outputs that the models have produced are examined. The latter three constitute the necessary information that computer scientists need to know despite their limited understanding of biology. Five omics levels are examined: genomics, transcriptomics, proteomics, metabolomics, and epigenomics (Table 1). The information that is included in this work could guide computer scientists to deeply understand omics data and data representations for deep learning-based predictive modeling and subsequently, make substantial contributions to life science research.

The rest of the paper is structured as follows. In Section 2, the foundational concepts are presented, and the adopted research methodology is analyzed. Section 3 offers a detailed exposition of the most prevalent types of biological data that are used to train DL models. The main focus is on the data and the representations, while the information about the DL architectures and the tackled problems are also provided. In Section 4, some of the major challenges in applying DL to biological data are discussed. Finally, Section 5 concludes the paper and presents the future prospects in the field.

## 2. Materials and Methods

In this section, the foundational concepts are presented. The types of omics data are reviewed, and the basic tasks that omics data are used for in conjunction with DL are summarized. The research methodology that is followed in this work is also presented.

### 2.1. Foundations

#### 2.1.1. Omics Data Types

Biological systems can be examined from multiple different aspects. An organism’s DNA sequence may be examined, and this is the part that contains all of the structural and functional information that is needed to sustain it. The chemical footprints of the metabolic functions that take place inside a cell can be analyzed, as well as the substrates and byproducts of cell metabolism. These different aspects are what the term omics refers to. However, in the upcoming section, the various data types that are associated with each of the most important omics levels (Table 1) are examined in depth; in this section, their biological significance is briefly explained.

*Genomics*. Genomics deals with the information that is encoded in the DNA sequence of an organism, and studies have been conducted on how different pieces of this information, which are called genes, influence the organism. Genes may play a role in the structure of an organism, or a cellular function, or they may merely regulate other genes in complex relationships, thereby forming gene regulatory networks. It is a single continuous DNA string that contains these different pieces of genetic information, the genes, which is like a file of computer code containing numerous functions. Each organism carries its own genetic material and, given that biological phenomena map the information that is encoded in the DNA, patterns that are mined from the genomics data can be applied to multiple predictive modeling tasks.

*Transcriptomics*. Every single cell of an organism contains a ’carbon copy’ of the same DNA string, yet not every cell uses it the same way. A type of selective usage takes place, with some parts of the DNA being transcribed and used by the cell, while other parts are left out. This selective DNA transcription and deployment explains why the different types of cells can assume different shapes and functions even though they all share the same DNA or why a biological system responds dynamically to its outer environment. Transcriptomics indicates the parts of its DNA that a cell has used. It indicates which genes were expressed, and to what degree they are done so.

*Proteomics*. Every biological function is performed by the proteins, and every type of cell and tissue is built of proteins. These are the building blocks of living systems. When one is studying proteins, the interest may be either in their amino acid sequence or in their three-dimensional structure; both of these are modalities containing the information on how a protein functions or interacts with other proteins.

*Metabolomics*. The chemical compounds that are produced or utilized during cell metabolism are called metabolites. Different types of metabolic processes are involved in virtually every biological event, and the different processes give off distinct chemical byproducts. Thus, the metabolomics data have interesting potential for being used for predictive tasks.

*Epigenomics*. Other than the sequential order of the nucleotide characters, the DNA string of an organism has additional layers of organization. These are provided by its three-dimensional structure, the time-dependent alterations of that structure, and certain chemical modifications affecting the DNA. These structural elements, which are the subject of study of epigenomics, have a massive effect on the biological functions, and thus, they carry information that is not present at the genomic level.

*Multi-omics*. Each omics type can only account for a certain range of biological phenomena [21], and the single-omics data reveal information only about one aspect of a system [10]. For example, even though the information that is used to create proteins is stored in genomic sequences, a protein-coding gene can give rise to different proteins due to alternative splicing; therefore, the DNA alone does not reveal everything about the proteins [22]. Two or more omics types can be combined for a multi-omics analysis, with each individual omics type providing the predictive model with unique, useful information.

#### 2.1.2. Tasks Solved with Omics Data and Deep Learning

The tasks where omics data are being used in conjunction with DL could fall into four distinct categories, as shown in Table 2:

1. *Biological*: Tasks concerning the prediction of biological attributes, such as predicting which parts of a DNA sequence will acquire a certain property, or the discovery of new biological knowledge, such as clustering genes into functional groups that interact with each other.
2. *Biomedical*: Tasks related to understanding disease, such as identifying genes that are affected by a certain pathogen, or making health-related predictions, such as predicting whether a patient is low- or high-risk.
3. *Drug Research*: Data-driven pharmaceutics applications include predicting the effect that a chemical agent will have on cells or inferring which chemical agent was applied to a biological sample.
4. *Bioinformatics*: Efforts to automate some of the low-level bioinformatics procedures that deal with data engineering pipelines. Tasks such as performing peak integration from mass spectrograms fall into this category.

In the following section, the types of omics data and data representations that are used in the literature to train the deep neural networks are examined in detail. Yet, in this introductory section, a summary table of the most common data types and representations, Table 3, is provided. The data types can be text strings, numerical tabular, and numerical (1D vector) time-series.

The numerical data can be fed into the neural networks as they are, or this can be performed after a simple pre-preprocessing procedure, such as normalization. The character string data can be represented with a variety of methods, such as, both domain-specific and general Natural Language Processing (NLP) techniques. The sequential string data might constitute hundreds or thousands of characters for each sample, and the numerical tabular data can have thousands of features. This complexity, reflecting the complexity of the biological systems that the data were captured from, explains the trend towards using deep neural networks for predictive modeling. Other than the generic predictive and regression tasks, DL is also used for knowledge discovery, e.g., training a model for classification purposes, and then, investigating the model to identify which features and combinations of features will determine the outcome.

From the review literature on the topic of DL in computational biology, the most suitable research articles to introduce the subject to a beginner are those of Zou et al. [20] and Angermueller et al. [16]. For the general overviews on DL, ranging from beginners’ articles to thorough expositions of the various models and techniques, valuable insights can be found in [23,24,25,26].

### 2.2. Research Methodology

**Figure 1:**
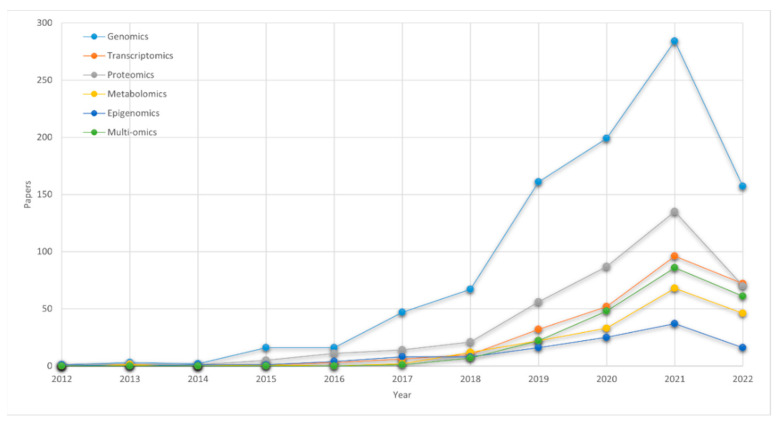
Number of papers combining DL and omics data over the last decade (statistics range to 19 August 2022).

**Figure 2:**
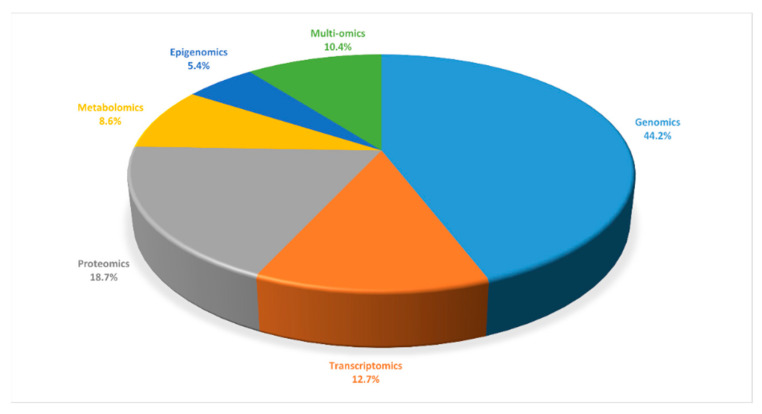
Papers combining DL and various omics types in the referenced literature over the last decade (statistics range to 19 August 2022).

The adopted methodology consisted of two steps. First, a Scopus search was conducted by using the combinations of terms “deep learning”, “genomics”, “transcriptomics”, “proteomics”, “metabolomics”, “epigenomics”, and “multi-omics”, i.e., “deep learning genomics”, “deep learning transcriptomics”, etc. The search was conducted on 19 August 2022, and it covered the last decade, i.e., from 2012 to 19 August 2022. The statistics from the search results are illustrated in Figure 1 and Figure 2; Figure 1 presents the number of papers, combining DL and omics data, while Figure 2 proportionally illustrates the involved omics types in the DL-related bibliography. It should be noted here that the quantity of genomics data and DL applications has grown greatly during the COVID-19 pandemic, from 2019 to 19 August 2022 as they are essential public health tools; the genomic data have supported the global health response, enabled the development of testing methods, and helped towards the timely tracking of novel SARS-CoV-2 variants. The latter is clearly illustrated in both Figure 1 and Figure 2. Second, the results were filtered both for their general relevancy and, most importantly, for the unique requirements of the proposed study. Since the focus is on introducing non-biologists and non-bioinformatician DL experts into the field of computational life sciences and non-DL experts biologists to the DL paradigm, the papers that were selected shared explicit information on the used data, how the data were represented and preprocessed, and they emphasized the ways in which the DL was applied. Therefore, the reviewed papers basically contain the meaningful information about the data that computer scientists would need to know in order to contribute to this research field.

## 3. Omics Data and Deep Learning

In this section, the applications of DL for different types of omics data are reviewed. The indicative literature was selected as they cover the state-of-the-art methodologies in the popular applications of the most recent years.

### 3.1. Genomics

**Figure 3:**
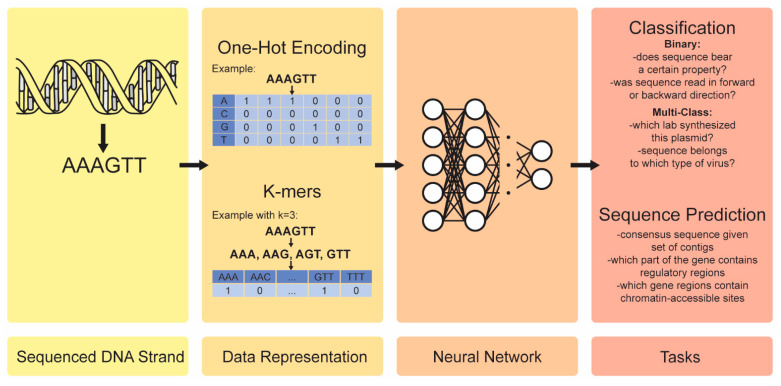
High-level view of DL applied to genomics. DNA sequences are represented either with one-hot encoding or k-mers, and they are used to train DL for certain tasks.

The raw material of genomics research is the DNA sequences which, from a computational point of view, are strings that comprise four characters: A, C, G, and T. To serve as the input data for the predictive modeling, the DNA sequences are usually represented in either of the two following ways:

1. One-hot encoding: a 2D matrix with four rows (each for one of the four characters) and a number of columns that is equal to the DNA string length. For each column, the character that was found in that position in the DNA sequence gets value one, while the rest of the three rows are given the value 0, as it is shown in Figure 3.
2. k-mers: A vector is generated, representing the possible permutations of the four nucleotides for a user-defined k (e.g., for k = 3, the permutations are AAA, AAC, AAG, …, TTC, TTG, TTT). Each element of the vector takes the value one if the permutation is present in the string; otherwise, it takes the value 0, as it is shown in Figure 3.

In [27], the DNA sequences of the synthetic plasmids were used to predict the lab of origin of the synthetic DNA. After the one-hot encoding, the sequences were used to train a CNN, which correctly identified the source lab 48% of the time, and for 70% of the time, the true origin appeared in the top-ten predicted labs. DL-based genomics techniques, which use DNA sequences as training data, have lately been proposed for the primer construction for PCR tests to detect COVID-19 [28] from human samples. The problem was formulated as being both of the multiclass (what type of virus) and binary class type (COVID-19 or not). A CNN achieved 98.73% accuracy for the binary problem. One-hot encoded DNA strings were also used for a DL-based approach to automate part of the sequencing pipeline itself [29]. The DNA sequencing methods produce clusters of short variations of the DNA substrings called *contigs*, which are then aligned to a reference genome to generate the consensus sequence. However, this is a computationally expensive process that combines dynamic programming and demanding algorithms to navigate the intractably vast search spaces. Researchers have automated this process via DL using contigs as the input data and the consensus sequences as the labels. After training a Bidirectional Long Short-Term Memory (BiLSTM) model that scanned the contigs with a window of size three, the research team in [29] predicted the consensus sequences with up to 99.81% identity with the ground truth, thereby surpassing all of the state-of-the-art models, including a tool that was released by Oxford Nanopore, a leading company in sequencing technology.

Another example of deploying DL to solve a common bioinformatics challenge was reported in [30], where the genomic sequences were classified as to whether the sequencing machine was reading in the forward or the reverse direction. The conventional bioinformatics techniques that were used to infer it were quite demanding, and they required a reference genome, which is problematic when one is sequencing a new organism for which there is no reference genome available. The input data were long reads of the DNA, which are represented as k-mers, with k ranging from one to five. A DNN and a CNN were trained, with the CNN performing slightly better than the other one. For validation, the researchers clustered the similar sequences together and performed a type of majority voting, where the majority of the orientation predictions of the clustered sequences were applied to all of the sequences in the cluster, and thus, the models predicted correctly up to 96.2% of the human reads and up to 98% of the S. cerevisiae (yeast) reads.

In [31], a CNN was trained on one-hot encoded DNA strings to predict the gene regulatory regions, i.e., the regions within a DNA sequence with activated genes. The annotations were a time-series that signified which parts of the sequences were inactive and which were active above a certain threshold, which were represented by vectors of 0 s and 1 s, respectively. The researchers observed that the window and stride size of the convolutional layers had a major importance in the model’s effectiveness.

Desai et al. [32] used the DNA strings themselves without one-hot encoding them, and passed them through an embedding layer to extract the representations. The task was to identify the bacteria from environmental samples. The classification was hierarchical, with bacteria being classified on three taxonomic levels: family, genus, and species. For comparison, the researchers trained a Recurrent Neural Network (RNN), an LSTM, a BiLSTM, a CNN, and a Combinatorial CNN. The LSTM outperformed the others in the family-level classification, with it having a 91.24% accuracy, and the BiLSTM had the best performance for the genus and species levels, with it having 85.63% and 70.78% accuracies, respectively.

Tahir et al. [33] combined the one-hot encoded DNA strings with codon composition tables to predict whether the sequences exhibited a certain property, namely, whether they contained N6-methyladenine sites or not. Codons are k-mers with k = 3, and the number of possible permutations of the four nucleotides for k = 3 is 64. The codon composition vector of a DNA string is a vector with a length = 64, where each position takes a value of one if the corresponding codon is present in the DNA string, otherwise, it takes a value of 0. The architecture that was used consisted of a CNN employing the one-hot DNA sequence. Its output vector was then concatenated with the codon composition vector, and it was fed into a dense layer for the classification to be performed. The latter approach achieved an accuracy of up to 98.05%, and it surpassed other reported methods by at least 2%.

In a similar way, Phuycharoen et al. [34] combined the one-hot representation with the codon composition tables to train the CNNs to predict which sequences contained the TF (Transcription Factor) binding sites in a 3-class problem (increased binding, decreased binding, and non-differential binding). The DNA sequences were also combined with a dinucleotide composition matrix in a two-tier classification system that firstly predicted whether the DNA contained promoter regions and then, in the case that it did not, it predicted whether the regions were strong or weak promoters [35]. Dinucleotides are k-mers with k = 2, and a dinucleotide composition matrix is a 2D matrix with rows corresponding to the samples, columns corresponding to all of the possible 2-nucleotide permutations (i.e., AA, AC, …, TG, TT), and data that are the normalized frequency of each dinucleotide of each sample. First, a CNN employed the one-hot encoded DNA sequences, its output concatenated with the dinucleotide frequency matrix, and a dense layer classified the resulting vectors as to whether they contained a promoter or not; for those vectors which did, the same pipeline took place, with a second CNN taking the one-hot DNA sequences, concatenating its output with the dinucleotide frequency matrix, and a dense layer classifying whether the promoter was weak or strong. The proposed approach surpassed the accuracy of the previous benchmark methods by 2–10%.

K-mers have been used not only in composition matrices, but also in their preliminary form, as they are vectors of strings of length = k, resulting from the sliding window over the original sequence (e.g., with k = 3, TACGG becomes {TAC, ACG, CGG}). These lists of strings are treated as a corpus of texts, and natural language processing techniques are applied to learn the word embeddings. In [36], the k-mers were transformed with Glove embedding method [37]. A hybrid CNN–BiLSTM was trained to predict which DNA sequences contained the chromatin-accessible sites, thus signifying that these sequences play functional roles in a biological system. The hybrid model performed better than its component models did individually, and its accuracy surpassed that of the other previously used methods by 1–7%. Guo et al. [38] compared the Glove and word2vec [39] embeddings of the k-mers. The task was performed in the same way as it was previously performed, and the sequences were evaluated as to whether they displayed chromatin accessibility or not. After training the hybrid CNN-GRU models that had an additional attention layer, the Glove embedding method was proved to be better than the word2vec one, and the overall performance of it was comparable to those of the other state-of-the-art methods. In [40], word embedding and FastText-transformed k-mers were used for the binary task of classifying the DNA sequences as to whether they contained, or belonged to, essential or non-essential genes. The used model was an ensemble of both of the shallow ML and DL models, comprising a k-nearest neighbors (k-NN) one, a random forest (RF) one, a support vector machine (SVM), a DNN one, and a CNN one. The model achieved 76.3% accuracy, 84.6% specificity, 60.2% sensitivity, and it was comparable with the other state-of-the-art methods, surpassing most of them.

In [41], an automatic framework, namely AMBER, which is used for designing CNNs in genomics was presented, and it was based on a novel Neural Architecture Search (NAS). The pathology type was encoded to be one-hot, which was the label. AMBER was applied to the modelling genomic regulatory features, and this resulted in the achievement of better predictions of the disease-relevant variants when they were compared to those of the basic non-NAS models. Li et al. [42] proposed a DL genomics approach, and they applied it to a multitasking classification of Alzheimer’s disease progression by identifying the novel genetic biomarkers that have gone unnoticed by the traditional genome-wide association studies (GWAS). The classification accuracies achieved up to 99.44% by using the proposed DL genomics model. Chalupová et al. [43] developed an easy neural network tool for genomics (ENNGene) to bridge the gap between the need for DL models in genomics and the limited ability of researchers in the field to develop one. ENNGene could deal with multiple input branches, and it could be fully customized by the user. The results of using ENNGene were similar to those of the state-of-the-art ones.

### 3.2. Transcriptomics

**Figure 4:**
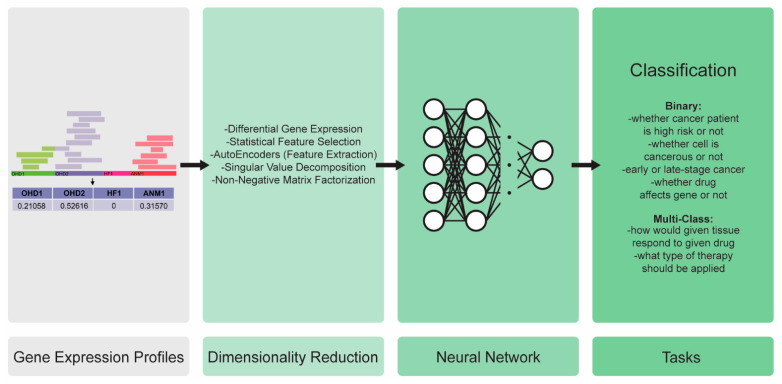
High-level view of DL in transcriptomics. The number of reads mapped to each gene during sequencing are normalized into gene expression profiles. These usually go through dimensionality reduction techniques and then serve as training data for various classification tasks.

Gene expression profiles quantify the activity of each gene within a biological sample. The more active the gene is, then the more it is transcribed into the RNA, and the RNA sequencing yields more reads that map to the active genes than they do to the inactive ones. The active genes have more reads. The formulas that were used to normalize these read counts do not focus simply on the numbers of reads, but also take into account the read- and gene-lengths. More details on the full transcriptomics pipeline can be found in [44,45,46]. The final results of the process, which were used as the data in the predictive modeling, are numerical vectors with values that are between 0 and 1, denoting how much each gene is expressed within a sample (Figure 4).

Transcriptomics data have occasionally been used for the completion of regression tasks, such as inferring a patient’s age from the gene expression profiles of the protein-coding genes [47], though most studies are tackling the classification problems of this. Being exceedingly high-dimensional with them having thousands of features, the gene expression data are more useful and tractable after a treatment with the dimensionality reduction techniques. The most common methodologies apply feature engineering and feature selection. The feature selection of the transcriptomics data, which is referred to as the differential gene expression (identifying genes that are expressed differentially depending on the class), employs a number of domain-specific techniques [48,49,50].

The feature extraction and the dimensionality reduction techniques have been applied. In [51], the researchers set out to determine which method was optimal for the gene expression data, concluding that the DL-based extraction with the proposed DeepAE was the best when it was compared to the four benchmark models: singular value decomposition, k-sparsity singular value decomposition, sparse non-negative matrix factorization, and the previous state-of-the-art one, a domain-specific method that is called CS-SMAF [52]. The evaluation was performed by comparing the original with the reconstructed transcriptomics data using the Pearson correlation coefficient, the Euclidean distance, and the mean absolute error as the metrics for the comparison.

A tangible portion of the DL-based transcriptomics research has been conducted in the field of oncology; in [53], the gene expression profiles were utilized in a three-fold binary class task. Firstly, this was performed to predict the high-risk patients. Secondly, this was performed to predict whether the patient would survive or not. Thirdly, this was performed to predict their event-free survival, i.e., whether the patient would survive without experiencing repercussions and side-effects from the treatment. The DL architecture was comprised of two integrated models. A DNN took the original dataset, while an Auto- Encoder (AE) took the dataset after it had been feature-selected for the High-Risk class. The AE-extracted features were concatenated at some point and integrated into the DNN, which generated the multi-output binary-class predictions. In the comparative experiments, this architecture was shown to consistently outperform the RF and linear-SVM.

In [54], a simple binary prediction task was combined with unsupervised learning for the purpose of knowledge discovery. First, a DNN took cellular gene expression data and classified whether each cell was cancerous or not. The cancerous cells were singled out, and through a k-means clustering, they were grouped with the goal of discovering the novel cancer subtypes that were not present in the input data labels. Compared with other clustering methods, the k-means were shown to yield better more biologically meaningful results. Lee et al. [55] classified early and late-stage cancer from the gene expression profiles, and they observed that with this type of data, an increase in the number of input samples raised the probability of bias due to there being outliers. The solution that they proposed was to use statistics to determine whether the differences in the gene expression values among different types of cancer were statistically significant. Since the t-test could be sensitive to large outliers, they used the Wilcoxon rank-sum test. After that statistics-based feature selection, they trained a DNN, which yielded a 94.2% accuracy.

In [56], a nested classification task was tackled, and first, it classified the tumor type, and then, it classified the molecular tumor subtype; both of these problems were multi-class. The researchers normalized the gene expression data of each patient, applied a log2 transform, and performed a feature selection by comparing the median expression of each gene for the in-class samples with the out-of-class samples; the median was more robust for the outliers than it was for the mean. By applying ResNet, 1D-CNN, and 1D-Inception, they reached a maximum accuracy of 98.54% for the primary tumor type, and maximum accuracy of 83.5% for the molecular tumor subtype.

In [57], the gene expression profiles were combined with the splice junction data for the unsupervised discovery of novel cancer subtypes. The splice junctions are DNA subsequences (exons) that are left out during the transcription from DNA into RNA, thus changing the function of the gene. In this study, a frequentist estimate of the inclusion level of each junction in each sample was calculated, thus resulting in a matrix of the shape [649 patient samples] × [34.425 skipping exons]. Conventional clustering is problematic in high-dimensional data, hence, in the proposed work, an AE-based pipeline reduced the dimensionality, and the learned latent-space representations were then clustered. First, two stacked AEs (SAE) took the input data, one SAE handled the gene expression and the other handled the splice junction matrix. Then, their outputs were concatenated and fed into an AE that extracted the final features, which were then clustered through the k-means. Compared to PCA-based clustering, the AE-based clustering yielded better results and identified more clinically meaningful cancer subtypes.

Another field profiting from DL-based transcriptomics is drug research. In [58], the gene expression profiles of tissues that were treated with drugs were used. Each sample was labeled by the properties of the chemical agent that were contained in the applied drug, e.g., antineoplastic, cardiovascular, central nervous system agents, etc. As a biologically relevant type of feature engineering, the researchers used OncoFinder [59] to transform the gene expression data into a matrix representation of signaling pathway graphs. The matrix represented the regulatory interactions among the genes, with rows and columns signifying the genes, and the values indicating the up-regulation, down-regulation, or there being no effect. A DNN was trained with this data using the effect that a chemical agent has on regulatory gene interactions to infer the drug’s properties. The DNN was proven to be better when it was compared to an SVM. It should be noted that the model might have concluded in a novel discovery; however, a certain drug was misclassified, and its biological effects contradicted its human-annotated label, and after reviewing the relevant literature, the researchers proposed the chemical agent as a candidate for drug repurposing.

In [60], the gene expression profiles of both of the chemical agent perturbations and gene knockdown perturbations were used for predicting the protein–drug interactions from protein coding genes. Chemical agent perturbations took place by applying chemicals to the tissues, and the researchers monitored how the genome responded, and which genes were up/down-regulated. The gene knockdown perturbations consisted of removing a gene through the use of some gene editing technique and monitoring how the other genes responded. The data were in the form of real-valued matrices. For the chemical perturbation matrix, the shape was [number of genes] × [number of drugs], and for the gene knockdown matrix the shape was [number of genes] × [number of landmark genes]. The labels were binary, and they represented whether a drug affected a gene or not. Thus, by using the two types of input data, it was possible to detect both of the direct interactions, where a chemical agent directly affected a certain gene or the indirect interactions, where an agent affected the other genes which, in turn, affected the gene in which they were interested in. A DNN was used with an input layer that had two channels for the two datasets. Then, the channels were concatenated, thus producing the final binary classification with an accuracy of 90.53% and an F1-score of 86.38%. The model proved to be superior to Logistic Regression, Random Forest, and Gradient Boosted Tree.

In [61], the transcriptomics data were combined with the gene signaling pathways and chemical structures of the drugs for the research of drug repurposing, i.e., finding drugs that can be used for diseases other than that which they were originally developed for. The idea was to train the models with the effect that the drugs had on the gene expression to identify the drugs that were misclassified by the human annotators, and then compare the chemical structure similarity and drug effect on the gene signaling pathways. By finding similar drugs and evaluating how these approved drugs had been classified, hypotheses for the experiments and discoveries could generate. In that particular study, the gene signaling pathways were in a graph form, which were represented as a matrix of genes with topological weights. Through the in silico Pathway Activation Network Decomposition Analysis (iPANDA) algorithm [62], they were transformed into an activation score matrix of the shape [number of samples] × [number of signaling pathways]. The gene expression profiles were simplified by clustering them into groups of similar genes. The chemical structure data did not participate in the model training, but they were only considered when an interesting finding occurred or when a drug looked promising, whereas the chemical structure comparison identified the similar drugs. A DNN was trained for a 6-class prediction, classifying the drugs based on their therapeutic effect. The experimental tests showed that combining gene expression and signaling pathway data yielded better results than either one of the individual data types did alone, and the researchers have reported a new discovery, which is a strong candidate drug for repurposing, which is awaiting its experimental validation.

In [63], the unsupervised inference of the regulatory networks was conducted on transcriptomics and gene ontology data. The gene expression profiles of the cells that were treated with chemical compounds were concatenated with one-hot encoded gene ontology annotations, which consisted of the categorical information of the attributes of the genes [64]. The real-valued gene expression matrix was first thresholded and turned into a binary one, with a value of one if a gene was differentially expressed due to the chemical treatment, otherwise, it was given a value of 0. A Deep Belief Network (DBN) performed a hierarchical-style clustering, with the gene clusters having been seen as the modules of the gene regulatory network. The resulting findings were confirmed by the Gene Ontology data and a subsequent literature review, and the regulatory networks that were inferred from the DBN-based clustering had biological validity. The researchers highlighted that setting the parameters for the DBN was an empirical, trial-and-error process with no theoretical foundation suggesting beforehand which parameters would be optimal. The choice of using a DBN was made for its robustness to the noise. The k-means and hierarchical clustering, being distance-based, were affected by the randomness in the data, while the DBNs were better at finding the generalization beyond the noise. The goal was to decode the gene regulatory networks in an analysis pipeline that used the DBN as the starting point for the clustering, and then, the researchers continued with the statistical tests and the other non-DL techniques.

In [65], DL and joint supervised classification have been used to characterize the molecular changes that are correlated to Alzheimer’s disease (AD). The mapping of the cohort with a heterogenous diagnosis to the same transcriptomic space took place, and an unsupervised dimensionality reduction was applied to obtain a progressive trajectory that is associated with the severity of the AD. Finally, the transcriptomic data were applied to the model from different areas of the brain and blood monocyte samples to evaluate the reliability of the findings for different cohorts and tissues. In [66], a machine learning technique used the molecular characteristics of tumors to generate personalized therapies. A cohort from which the cell line gene expression data were gathered was employed, and they could be classified into two groups with different pan-drug chemotherapeutic sensitivity. The Boruta feature selection algorithm was used, and a neural network with 10 hidden layers classified the pan-cancer cell lines into the two groups with 89% accuracy. The results indicated that the cell lines with similar gene expression profiles had a comparable pan-drug chemotherapeutic sensitivity. Therefore, the comparable biomarkers could be used to select the effective drugs to increase the therapeutic reaction, while at the same time reducing the cytotoxic problem.

Gene expression datasets are sometimes used in conjunction with gene interaction graphs to train the Graph Convolutional Networks (GCN). In [67], the researchers wanted to classify the cell types from their gene expression. They procured the gene expression profiles for various cells, and then used them to construct a cell similarity matrix by measuring the cosine similarity among the expression levels of the different cells. That matrix was then turned into a graph for a GCN training procedure, and it included both labeled and unlabeled cells (semi-supervised). Training the GCN with this graph plus labeled the gene expression data resulted in a model that took a single-cell gene expression profile and was able to output a probability matrix, thus representing the probability that the cell belonged to a number of preset types of cells. The GCNs also contributed to drug research. In [68], the GCNs were used to predict the drug response. The model was trained with the gene expression profiles of the cells that received a treatment with drugs plus a graph with the interactions among the genes. In [69], the researchers trained the GCNs to predict the interactions among the genes that are associated with cancer in a search for the non-essential genes that could be targets for drugs. Drawing on the concept of Synthetic Lethality (SL), the researchers hoped that identifying, within a cancer cell, a non-essential gene that interacted with the cancer-causing gene, the non-essential gene could be targeted by a chemical treatment and the cancer cell would die without affecting the healthy cells. The SL interactions were sparse, and training ML models were prone to overfitting, but the researchers managed to accurately capture the relationships among the genes using a Dual-Dropout GCN (DDGCN) that used both fine-grained and coarse-grained node dropout techniques, thereby achieving state-of-the-art results.

### 3.3. Proteomics

**Figure 5:**
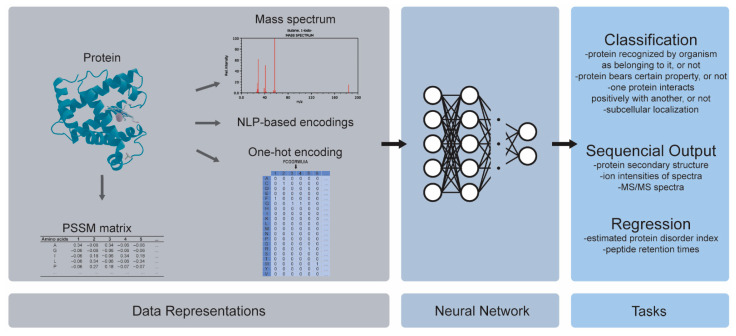
High-level view of DL in proteomics. Proteomics data can be represented in a variety of ways, e.g., mass spectra, NLP-based embeddings, one-hot encoding tables, or PSSM matrices. Then, neural networks can be trained for classification, sequence prediction, and regression tasks.

A protein or peptide sequence is a string consisting of the 20 amino acids, which are denoted with the letters A, C, D, E, F, G, H, I, K, L, M, N, P, Q, R, S, T, W, Y, and V. The simplest way to transform such a string into data for its predictive modeling is by using one-hot encoding, as shown in Figure 5. However, this is often coupled with Natural Language Processing (NLP) or with the representations that integrate domain knowledge into the data.

Natural language processing-based encodings may also be coupled with additional preprocessing. Borrowing from NLP, a number of studies have applied the skip-gram-based [39] encoding of protein sequences. In [70], the protein strings were transformed into a 20 × 15 matrix, where each amino acid in the sequence was a “word”, and the skip-gram encoded the 20 possible amino acids into a 15-dimensional space. The skip-gram encoding was followed by an embedding layer. A CNN took the embeddings and classified whether a protein could bind to the HLA class I regions of a genome, meaning that the protein would be recognized by the cell as belonging to the organism and therefore, no immune response would be triggered. The latter was a biomedical application that was useful for drug research and for understanding autoimmune disorders. The CNN performed comparably, depending on the dataset, with the other state-of-the-art models. In [71], another variation was proposed for the DL- and ML-based classifications of the protein sequences, which were tested with DNN and SVM, thus achieving high performance. The visualization of the encoded sequence space suggested that this encoding stored accurate information about the protein structure. The method encoded a sequence as a continuous-value vector that characterized both the sequential structure and physicochemical properties of the protein.

In [72], a novel natural language processing method that is called SeqVec (Sequence-to-Vector) was proposed and it was based on ELMo (Embeddings from Language Models) [73]. SeqVec was trained on unlabeled data, and it learned to predict, probabilistically, the next word/character given that it knew the previous words/characters. Learning these probability distributions was a similar process to that of understanding the syntax and grammar of the corpus, and the same word/character could have different embeddings depending on the words/characters that came before it. The researchers tested this novel embedding on a wide range of DL-based proteomics tasks, which fell into three different categories depending on the type of output that the DL model produced: (1) a sequential output, where the model predicted a protein’s secondary structure (a string of length equal to the length of the amino acid sequence, denoting the three-dimensional structure of the physical protein), (2) its classification, both 10-class (subcellular localization, i.e., whether the protein was located in nucleus, ribosome, membrane, etc.) and binary (whether water-soluble or membrane-bound), and (3) regression, where the model generated a continuous value for the estimated protein disorder. A DNN was implemented for the classifications, and a CNN was implemented for the sequential and regression tasks. Although the reported results did not surpass those of the previous state of the art approaches, SeqVec yielded a better performance than that which is obtained by using other encodings, and moreover, the SeqVec representation was produced faster.

In [74], an encoding method that is called MOS (Matrix Of Sequence) transformed a protein string into a 2D matrix with values ranging from 0 to 1. It resulted in the faster training of a model when it was compared to a number of other encoding methods. The trained model was a DNN that was used for the classification of protein interactions, and it yielded an accuracy of 94.34%. The amino acid sequences and physicochemical properties of the amino acids provided the necessary information to predict the protein structure [75], and a number of encodings were proposed to bring domain knowledge into the representation, thus integrating additional information into the sequential context of the data. Chen et al. [76] encoded the sequences with the Auto Covariance (AC) algorithm [57], which transformed the protein sequences into numerical matrices of the same dimensions, regardless of the sequence length. The 20 amino acids were grouped into seven physicochemical properties, and a normalized matrix was constructed to represent the information. Then, the matrix was transformed to fit into a user-configurable shape/dimensionality, ensuring that all of the sequences were represented with matrices of uniform dimensions. The researchers used this transformation to evaluate the host–pathogen protein–protein interactions (HP PPI), predicting whether two proteins had a positive or a negative interaction. A stacked denoising AE extracted the features, and following this, a dense layer for the final classification was applied. The proposed architecture surpassed the traditional machine learning models.

In [77], the proteins were classified as venomous or non-venomous, which is a task that is useful in antidote research. The protein representation was based on the Atchley factors [78], whereas each amino acid was represented with a numerical vector of five elements, corresponding to five physicochemical and structural properties (polarity, secondary structure, molecular volume, codon diversity, and electrostatic charge). Thus, each sequence was represented as a 2D matrix of shape 5 × [sequence length]. A Gated Recurrent Unit (GRU) was trained with the Atchley representations and the binary labels, and it surpassed the previous methods by up to 16% in its accuracy, and up to 22% in its F1-score. In [79], four different encodings of protein sequences and their combinations were used to train a CNN. The results compared: (1) a simple one-hot encoding of the amino acid sequence, (2) the Informative Physico Chemical Properties (IPCP), an encoding that quantified the physiochemical properties of the amino acids, (3) the Composition of K-Spaced Amino Acid Pairs (CKSAAP), which encoded the normalized frequency of the appearance of each possible pair of amino acids, without the two amino acids of each pair having to be next to each other, but with the k amino acids being interpolated between them [80], and (4) the Pseudo Amino Acid Composition (PseAAC), which used a set of discrete serial correlation factors, rather than the sequence’s actual composition. The researchers concluded that one-hot encoding that was concatenated with CKSAAP provided the best data representation, yielding 88.98% accuracy and an Area Under Curve (AUC) of 0.90, thus surpassing the previous methods. Ahmad et al. [81] classified the peptides as to whether they contained antifungal properties or not by concatenating the one-hot encoded sequences with three types of extracted features: (1) the Composite Physicochemical Properties (CPP), an encoding that described the amino acid composition and eight physicochemical properties of each protein, (2) the Quasi Sequence Order (QSO), whereas the sequential information of the protein was encoded using Grantham distance (chemical distance information) and the Schneider-Wrede distance-based matrix (distance based on physicochemical properties) among each pair of the twenty amino acids [82], and (3) a reduced amino acid alphabet, which was an abstract concept, consisting of different methods and proposals to represent a peptide sequence in a dimensionality that is smaller than that of a one-hot encoded sequence. The experiments demonstrated that the combination of the three extracted features performed better than any single feature did alone.

In addition to the amino acid sequence and the physicochemical properties, some researchers have aimed to enrich the proteomic data representations by integrating the evolutionarily similar information. The latter was done with a Position Specific Scoring Matrix (PSSM), a 2D matrix of dimensions [20 amino acids] × [protein length], as shown in Table 4.

The values of Table 4 were computed using the online PSI-BLAST tool [83], which searches the Swiss-Prot database and calculates, through multiple alignments, each sequence’s evolutionary similarity with the proteins that are from other lifeforms. On an abstract level, a PSSM can be seen as the location of a protein in the protein sequence space of all of the lifeforms that share a similar protein. Since one dimension of the PSSM is the sequence length, a transformation called PsePSSM (Pseudo-Position Specific Scoring Matrix) was developed so that the protein sequences of varying lengths could be represented by the matrices of uniform shape. Numerous variations of the aforementioned protein representations have been developed [84,85,86,87,88,89,90,91], and they share two common characteristics. Firstly, they integrate the domain knowledge into the data, which represent additional information that is not present in the amino acid sequences themselves; this information may consist of the physicochemical properties, or of the evolutionary similarities that are extracted through comparing the proteins across the lifeforms, thereby encoding some of these comparative insights into the data. Secondly, they often result in non-sequential data that can be utilized by any neural network or, for that matter, any conventional machine learning model.

When the protein and peptide sequences are not transformed into a representation that nullifies their sequential status, the DL models that are able to handle them are proven to be CNNs and RNNs. Wen et al. [92] used a genetic algorithm to find the optimal architecture of a hybrid CNN-Bidirectional GRU. The task that they performed was regression using a sequence to predict the peptide retention times (RT), which can be used as a quality control for the drug development. The researchers addressed the data shortage with a transfer learning procedure. First, they trained the model with various peptides and RTs, second, they trained it with the particular peptides of interest, and finally, they fine-tuned the pretrained model. Predicting the peptide RTs from a sequence was the focus of Ma et al. [93] who used a CNN with capsule layers [94], thus achieving a state-of-the-art performance. The CNNs with capsule layers were further validated by Du et al. [95], which resulted in it outperforming the conventional ML models in the binary task of predicting whether a protein was saliva-secretory or not. The study was part of a larger project of analyzing the cancer biomarkers in saliva, as saliva has certain advantages when it is compared to blood and urine for evaluating disease biomarkers. Armenteros et al. [96] asserted that a hybrid CNN with a BiLSTM was perhaps the best model for sequence classification, and this hybrid improved its performance with an attention layer. The hybrid CNN–BiLSTM processed the sequence, and the attention mechanism focused on the important regions within it. Both a binary and a 10-class task were studied, the binary task was the determination of whether the protein was membrane-bound or soluble, and the multi-class was a subcellular localization one, i.e., determining the protein’s location in relation to the cell (e.g., nucleus, cytoplasm, membrane, extracellular, etc.). The attention-augmented hybrid achieved 78% accuracy for the 10-class task, and 92% accuracy for the binary, thus outperforming the previous state-of-the-art one.

**Figure 6:**
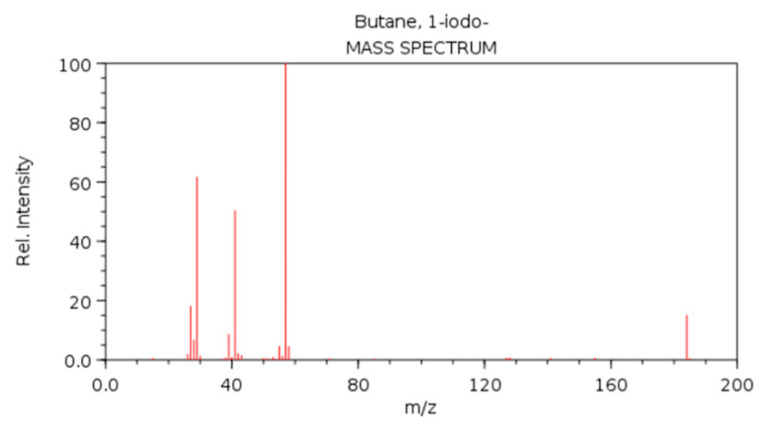
An example mass spectrum of Butane, 1-iodo-, formula: C_4_H_9_I [98].

In [97], the proteomics data were coupled with the GCNs for drug repurposing. The scientists drew from various datasets to create one large multi-relational graph of drug–protein, disease–drug, disease–protein, and protein–protein interactions. A CGN was trained to predict whether a drug and a disease could be associated, i.e., whether the drug could be used as a treatment for the disease. Another data type that is common in computational life science is Mass Spectrometry (MS) data. A mass spectrum, which is taken from any molecular structure, chemical or biological, measures the mass-to-charge ratio (*m*/*z*) of the ions that are contained in the sample, as shown in Figure 6. It is represented by a sparse 1D vector, denoting the relative abundance of the ions (y-axis, value in the vector) for each position in a discretized *m*/*z* spectrum (x-axis, position in the vector). Through MS, conclusions can be made regarding which molecules a sample consists of, and this can be used to determine its chemical composition.

Guan et al. [99] used one-hot encoded sequences to infer various tandem mass spectrometry (MS/MS) properties: (1) the ion retention time (iRT), which was a continuous value, (2) the survey scan charge state distributions, which were histogram-type vectors, and (3) the sequence ion intensities of the spectra, which was a one-dimensional time-series vector. In that multioutput determinations, the highest performance was reached with a BiLSTM, which comparatively surpassed the CNNs. In [100], a hybrid CNN and BiLSTM technique inferred the MS/MS spectra from one-hot encoded peptide sequences, thereby achieving state-of-the-art results. In [101], the same task was tackled with a seq2seq type of Residual CNN, with the difference that the one-hot encoded sequence was concatenated with a numerical vector of the monoisotopic mass of each amino acid in the sequence.

### 3.4. Metabolomics

**Figure 7:**
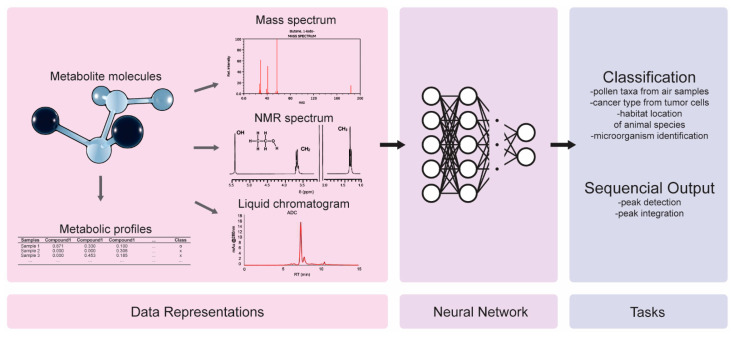
High-level view of DL with metabolomics. The metabolites in biological samples are represented as either various types of spectra or as metabolic profiles. Neural networks can then be trained for classification and sequence prediction tasks.

Most of the metabolomics data types fall in the time-series category, which refers to the MS spectra (Figure 6), the Nuclear magnetic resonance (NMR) spectra (Figure 7) the liquid chromatograms (Figure 7), etc. These techniques elucidate the chemical composition of the samples, resulting in histogram-type plots, which are represented as one-dimensional vectors.

With DL-based metabolomics, the classification of the biological systems does not rely on the genetic makeup and activity, e.g., the genomic sequence and gene expression, but on the chemistry of it. Probing into the chemical composition of the metabolites, the byproducts of cell metabolism, provides information and data for the classification tasks, thereby identifying the biomarkers for various traits or diseases, as well as for drug research. 

Regarding the task of processing the spectrometry data for DL model training, Akyol et al. [102] recommend two preprocessing practices: (1) replacing the missing values with some very small values, which are normally half of the minimum positive value of the data, and this was performed because the missing values probably express the metabolites whose levels were too low to be detected, and (2) applying a quantile normalization to reduce the variability among the different samples. Klimczak et al. [103] used NMR spectra to identify the different taxa of pollen from the metabolites that were contained in air-sampled pollen extracts. The latter could provide useful information to patients who are allergic to specific types of pollen, and predict the allergy outbreaks during the spring, a capability that is particularly valuable for the areas where a tangible part of the population is afflicted by a pollen allergy, such as in Central Europe. Previous studies tried to classify the pollen by using carefully prepared samples that contained purified pollen in very high concentrations. That approach aimed to be realistic and deployed a data-driven approach to identify the pollen species from natural samples. A CNN learned to classify the NMR spectra of three species of pollen, and it achieved an accuracy between 86% and 93% depending on plant type. The MS spectra of tumors were used for a six-class cancer type prediction in [104]. A number of spectra were taken for each tumor, with the range of ion *m*/*z* scanning and the number of peaks varying in each spectrum. In order to make the spectra uniform, the researchers performed binning on the *m*/*z* range, and they summed up the peaks that fell within each bin. A DNN was trained with the resulting vectors, thus yielding significant performance gains over the traditional ML models. In that study, the batches of spectra were integrated manually into a single spectrum; however, in [105] the integration was achieved through the use of DL. The labels were hand-crafted spectra which were made by human experts from batches of spectra of the same peptide, where the peaks in the different spectra rarely coincided in the same regions on the x-axis. A BiLSTM took the spectra and learned to perform peak integration. The quality of the model’s output, after adequately training it, was slightly lower than that of the human annotators. 

DL has been also deployed for metabolomics data annotation to automatically detect the peaks from spectra and chromatograms. In [106], the peak detection in LC–MS (liquid chromatography—mass spectrometry) data was learned from three-class labels, where each position on the input vectors was labeled as peak, no-peak/noise, or uncertain. The peaks were scaled to a maximum value of one to limit the selection bias towards more abundant peaks, and a custom, simplified U-net was trained to segment the LC–MS images according to the three classes. The model achieved an intersection-over-union (IoU) of 0.88 for the separation regions and 0.85 for the peak regions. A U-net was also employed in [107] for chromatogram peak detection. The researchers devised a method to generate synthetic chromatograms and annotations to train the U-net model, which identifies the peaks with a higher accuracy than the human experts did.

Raw spectra and chromatograms may also be turned into metabolic profiles, as shown in Table 5, representing the chemical composition of each sample. The columns represent various compounds, the rows represent the samples, and the values quantify the relative abundance or concentration levels of each compound in each sample.

Date et al. [13] used the metabolic profiles of yellowfin goby fish that were collected from rivers across Japan. As it was more of a proof-of-concept study, the researchers simplified the task by keeping it as a binary classification problem, and they trained a DNN to detect whether the fish originated from Kanto or not. The model reached a 97.8% accuracy, performing slightly better than RF and SVM did. Hypothesizing that the organisms of the same species feeding in different locations would vary in their metabolic profiles accordingly, the researchers explored the possibility of inferring the habitat location via the DL-based metabolomics. In a similar Japanese study [108], the researchers used metabolic profiles to infer the physiology of the subjects. They also applied the study to fish of multiple species throughout Japan after noticing that fish size was highly correlated with metabolite composition. Using fish size as the dependent variable, they trained an ensemble of DNNs to perform regression. Each DNN was trained on bootstrapped data (samples randomly picked with replacement), and additionally, each DNN was trained on a random subset of the features/variables. The best models were combined for the final prediction, and the final result was always better than that which the regular DNNs could produce, and depending on fish species, it was comparable with the RF and SVM ones. Guo et al. [109] used the metabolic profiles of chronic kidney disease (CKD) patients and healthy control patients to identify the biomarkers for CKD, and this made it possible to predict CKD in its early stages. The labels consisted of five CKD stages plus one class for healthy individuals. After applying the feature selection with a Lasso regression [110] on the dataset, a DNN and a CNN were trained. The DL models ended up performing worse than the RF did, which was attributed to the extensive feature selection; the models were trained on only five out of tens of thousands of features. A comparable performance was achieved by using ML and DL when dealing with low-dimensional datasets and a reasonable (not too high) number of samples.

In [111], a platform combining metabolomics and DL was applied for pathogen and spoilage microorganisms identification. A CNN model of three potential biomarkers for Listeria Monocytogenes was developed, reaching predictions of up to 82.2%. Moreover, 29 metabolites were identified, and six common Listeria species were distinguished in hierarchical cluster analysis. Finally, the binary and multiple CNN classifiers identified Listeria Monocytogenes and other pathogens, with an accuracy of 96.7% and 96.3%, respectively. In [112], the metabolic profiles of breast cancer tissue samples were used to classify the estrogen receptors as positive (ER+) or negative (ER-). After the quantile normalization, the log transform, and mean centering procedures, an AE was pretrained to reconstruct the data, which was then converted into a DNN and trained for the binary classification. The method reached an AUC of 0.93, and it surpassed the traditional ML models. The end goal of the study was not the binary classification itself, but the elucidation of the biological functions and metabolic pathways that lead to different types of cancer. Starting from the binary classification, the researchers ranked the features in terms of how much they contributed to the outputs, thus identifying the important metabolites. With the database searches, they mapped these metabolites to the chemical pathways and enzymes. They also used gene expression data, thereby locating the genes that were expressed differentially in the two cancer types, and they analyzed these data to infer the gene metabolite networks. Thus, DL was used as a steppingstone, a component of a wider research methodology, to provide deeper biological insights.

### 3.5. Epigenomics

**Figure 8:**
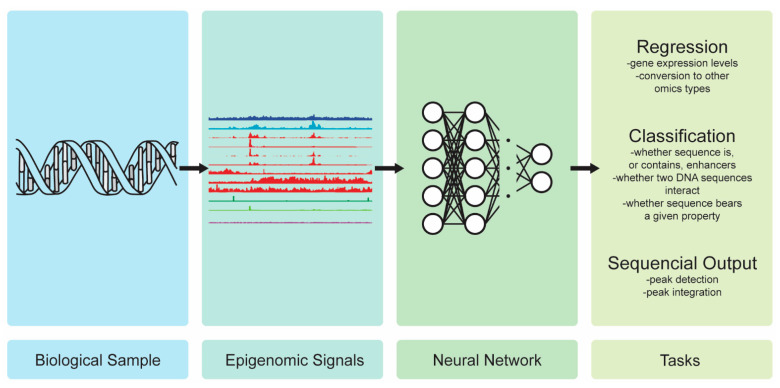
High-level view of DL applied to epigenomics. DNA sequences are represented as epigenomic signals, and they are used to train DL applications for certain tasks.

Epigenomic modifications and properties such as histone modification, DNA methylation, and chromatin accessibility can be seen as an additional level of information on top of the genomic level, thus adding to and modulating the information that is contained in a genomic sequence. A blood cell, neuron, or sperm cell of an organism all carry the same DNA sequence in their nucleus. It is the physical 3D structure and epigenetic modifications that define whether the DNA strand will result in a blood cell, neuron, or sperm cell. Predictive modeling, depending on the task that is performed, may require information that is not present in the DNA sequence, but this could be extracted from a higher level of biological organization, whether this is transcriptomic, proteomic, metabolomic, or epigenomic. From a computational point of view, the epigenomic data are comprised of time-series signals, with the x-axis spanning the length of an assorted DNA sequence, thus revealing which parts of the sequence have received a certain modification and bear a certain property. These signals are represented as vectors, which are either real-valued or binary. Figure 8 illustrated the high-level view of DL with epigenomics.

In [113], a differential gene expression was inferred from the histone modification profiles of a gene in two cell types. The same gene was expressed differently in the different cells; that differential expression was not represented in the DNA strings, but it could be extracted from the epigenomic data, which is the additional layer of information that is about which modifications were applied to the DNA sequence. Each sample in the dataset consisted of two vectors, representing the histone modification profiles of a gene in two cell types. Each vector showed the amount of histone modification taking place in the gene throughout its sequence length. A multi-head LSTM with an attention layer was trained for the gene expression levels regression, whereas a separate LSTM was trained for each input vector. An attention layer step followed this process, then an embedding layer step followed this. The embeddings were concatenated and fed into an LSTM and an attention layer before the final prediction was made. The model significantly outperformed the RF regression and the SVM regression, and it slightly outperformed the previous DL-based state-of-the-art methods. Similarly, histone modification profiles were used in [114] to detect the genomic sequences that were enhancers (DNA sequences that stimulate gene expression) or those that contained them. The dataset contained vectors that spanned the length of a DNA string with values signifying how much that a histone modification was applied at each point in the sequence. Each sample had a number of such vectors, which were stacked one over another, and each vector documented a specific histone modification. A hybrid CNN–LSTM was trained on a variety of datasets, and this reached an accuracy ranging from 84.8% to 98.0%, which was comparable to, and mostly surpassing, the previously applied DL models. In [14], the genetic predisposition for type-2 diabetes was detected with the help of a U-net. Part of a larger project, which was aimed at exploring the mechanisms and genetic pathways contributing to type-2 diabetes, DL was deployed to infer, with a signal from a few samples, what the signal from many samples would look like. The epigenetic information came in the form of ATAC-Seq signals, which were time-series vectors that were taken from type2 diabetic cells. The ATAC-Seq data were normally taken from multiple cells, the peaks were then integrated, and the signals were aggregated into one. The latter part was highly problematic when they were dealing with rare cells. The researchers trained a U-net to take the aggregate the ATAC-Seq signal of 28 cells, upscale it, and predict the aggregate signal of 600 cells. The result was subsequently used in the following stages of the research project.

ln [115], the genomic and epigenomic information was combined to predict whether two DNA sequences interacted with each other. The genomic information came in the form of the DNA sequence pairs that were one-hot encoded. One string always belongs to a promoter, and the other belongs to an enhancer, and a binary label signifies whether the promoter and the enhancer interact with each other. Regarding the epigenomic information, each genomic sequence is associated with a stack of vectors, representing the epigenomic features (e.g., CpG methylation, histone modification, etc.). The vector length represents the length of the DNA sequences that are divided in bins, and the values represent the degree to which an epigenomic property applies to each part of a sequence. Three models were deployed in [115]: a CNN, a CNN with an attention layer, and ResNet. All of the models followed a general architecture of starting as two separate models, with one taking the DNA sequences and the other one taking the epigenetic information, and later, the outputs of the two models were concatenated and fed into further dense layers to produce the final predictions. The experimental tests showed that integrating sequences with the epigenomic data yielded better results than using just using one input, and the epigenomic features were generally more informative than the DNA sequences were. The train–test split of the data was performed not randomly, but by chromosome; one chromosome provided the data for the training, and another provided the data for the testing. When dealing with enhancer–promoter interactions (EPI), random train-test splits may lead to one overestimating the model’s accuracy as there is great redundancy of the enhancer and promoter sequences and they may be present in both the training and the test sets. In [116], an attention-based DL model was developed, namely eDICE, to impute the epigenomics tracks. The model reported a state-of-the-art overall performance, and it was able to correctly predict the individual and cell-type specific epigenetic patterns.

We have examined the tasks, such as predicting differential gene expression and enhancer–promoter interactions (EPI), whereby more useful and discriminative information is found in the epigenomic than that which is found at the genomic level. However, the epigenetic data are generally hard to acquire, and the capability to predict the sites with epigenetic activity from the DNA sequences is being pursued [117]. Such predictions give direction to experimental research or, ideally, they can even be a substitute for the experimentally produced epigenetic data. In [15], a CNN takes the DNA sequences from different cell types and detects the histone modifications in an effort to identify the genes and pathways that correlate with aging. The volume of the DL-based epigenomic studies is still relatively low, but as the experimental techniques become cheaper and more mature, the production and availability of the epigenomic data increases, as shown in Figure 1. With mentions of a late “epigenomics data deluge” [118], the research in this area is expected to take off.

### 3.6. Multi-Omics

**Figure 9:**
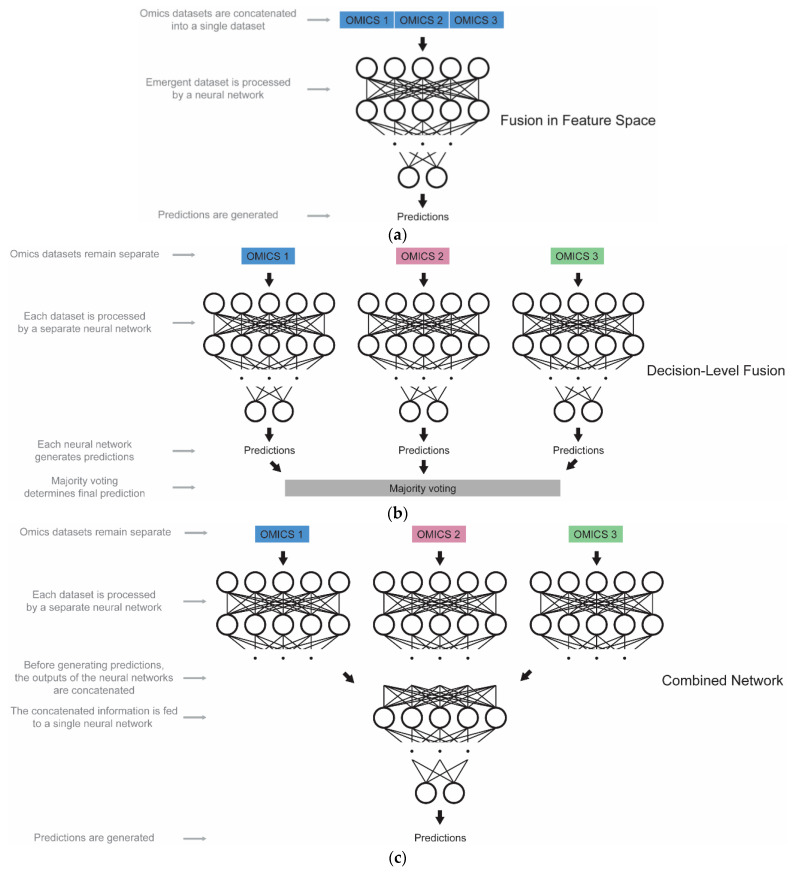
DL-based architectures for multi-omics data integration; (**a**) Omics data are preprocessed into a uniform type, then fed into a model; (**b**) A separate model generates predictions from each omics, which is followed by majority voting; (**c**) A multi-view model, with a separate neural network that takes each data modality, wherein their outputs concatenated and fed into further dense layers.

Any two or more of the five different types of omics data that have been previously examined, e.g., genomics, transcriptomics, proteomics, metabolomics, and epigenomics, can be combined for predictive modeling tasks, thus taking advantage of the unique information and patterns that are encoded in the different levels of biological organization. By learning from the multi-omics data, DL extracts the informative patterns from one biological level that are absent from another, thus exploiting the multifaceted information in a synergistic, holistic manner. The basic multi-omics data integration architectures that are used for DL model training are the following three:

- Different data modalities are preprocessed into a uniform type, then they are concatenated and fed into a model (Figure 9a).
- A separate model is trained for each data modality, their predictions are aggregated, and this process is followed by majority voting (Figure 9b).
- A multi-view model is used, starting as a separate model for each data modality, and then the outputs of these models are concatenated and fed into further dense layers, thus leading to the final prediction (Figure 9c).

Due to their capacity to learn the representations at different levels of abstraction through their hidden layers, DL models are particularly useful for modeling complex multi-omics data [119]. They are employed in multi-omics tasks, even as a small part of a pipeline that uses a plethora of computational tools. For example, in [120], DL was used only for feature extraction and a multi-omics integration stage of a research project aiming to achieve the unsupervised identification of cancer subtypes and the classification of new patients into these subtypes with the end goal of providing better patient care. The data came from hepatocellular carcinoma (HCC) patients, and this included genomic, transcriptomic, epigenomic, and clinical data. K-means clustering assigned labels to the data, and an AE extracted the features. The feature selection took place via the Cox PH (Cox proportional hazards) [121], and the ML models learned to classify the new samples according to the k-means-identified classes. In [122], a similar pipeline used RNA expression, miRNA expression, DNA methylation, and clinical data from cancer patients who were labeled by k-means clustering. The features were extracted through the AEs and selected through the Cox PH. Then, the ML models classified the new samples, and the statistical analysis was utilized for a biological insight, thus elucidating a differential gene expression in the cancer patients.

In [123], a wider variety of data was involved: mRNA expression, miRNA expression, protein expression, DNA methylation, somatic mutations, Copy Number Variations (CNVs), and clinical data. The DNA methylation data come in tabular form (Table 6), with rows being the samples, and columns being the sites in the genome of the organism that the samples were taken from, while the values show the amount of methylation that was applied to the genome on these sites. The somatic mutation data (Table 7) consists of the binary vectors that are of length equal to the number of the genes that are being evaluated, whereby the authors applied a value of 1 when the gene was mutated, and 0 otherwise. The CNV data come in the form of a table of shape [number of patients] × [number of genes], with the data taking one of three possible values (−1, 0, 1), showing whether for each gene the patient has no gene alteration, or whether they have some additional DNA regions that were copied, or whether they have regions that were deleted (Table 8). The same architecture as that which was used for the AE, the Cox PH, the k-means, and the ML-based classifications was implemented, with the researchers demonstrating that the multi-omics data yielded better results than single omics data did.

In [124], the same process was applied for ovarian cancer using a denoising AE instead of a regular or a stacked AE for the feature extraction procedure, which was followed by a feature selection procedure through logistic regression. In [125], the feature selection was implemented with a traditional statistical analysis, a regular AE that extracted the features, and an RF that classified new samples after it was trained on the labels assigned in an unsupervised manner. In [126], the feature extraction phase was implemented with two AE-based strategies using mRNA expression and DNA methylation data from Esophageal Squamous Cell Carcinoma (ESCC) patients. The first one was an early-fusion strategy, where the two omics datasets were concatenated and fed into a regular AE. The second one was a joint multi-modal strategy, where a distinct layer took each dataset, and their outputs were concatenated and fed into a common encoder layer. The outputs of the encoder layer are forked out into two distinct layers. The two distinct output layers learned to reconstruct the two datasets. The k-means clustering, which was based on the surviving rates, yielded two classes of high and low survival probability, the analysis of variance (ANOVA) selected the most important features, and an SVM learned to classify the data, thus showing that the joint multi-modal strategy led to better performance than early-fusion one did.

DL may also play a more central role in the multi-omics modeling tasks, as well as in cancer research. In [127], the classification of breast cancer subtypes was conducted using a CNN that took gene expression data and another CNN that took CNV data, and their outputs were then concatenated into additional dense layers that generated the final prediction. The model yielded a 79.2% accuracy, thereby surpassing the shallow ML models, and the combination of two omics data produced better results when they were compared to those of the individual omics. In [128], four types of omics data were treated with two AE architectures. The data, which came from breast cancer samples, consisted of gene expression, DNA methylation, miRNA expression, and copy number variations (CNVs). The AE implementations were applied with all of the possible pairs of the four omics data, i.e., the datasets were not combined, but different pairs were tested to find the best pairing. The first AE architecture, which is called the ConcatAE, consisted of a separate AE for each omics type. The extracted features were concatenated and fed into a dense layer for the classification to be performed. The latter was performed for all of the possible pairs of the four datasets. The second architecture, which is called the CrossAE, took a single omics dataset as the input, but it took two datasets as the output, thereby, it tried to reconstruct both datasets from one. The extracted features were averaged element-wise, and they were fed into dense layers for the classification to be performed. The classifications were both binary-class and multi-class, and these were used to identify the breast cancer subtypes. The best arrangement was proven to be ConcatAE with DNA methylation and miRNA expression data.

In [129], cancer classification was tackled with a GCN by integrating the multi-omics data along with the PPI networks, and this surpassed the other baseline methods to which it was compared. In [130], the researchers used the multi-omics data of cancer patients to generate, via a Similarity Network Fusion (SNF) method [131], a graph of the patient similarities. Then, by using that graph and AE-extracted features of the original multi-omics data, they trained a GCN to classify the types of cancer. Thus, the model could identify the cancer not only by the multi-omics data of a patient, but also by taking into account the diagnosis of similar patients. Similarly, in [132], the researchers did not want to rely exclusively on the multi-omics data for the classification of the cancer, thus, by using Pearson correlation, they constructed a similarity graph between the samples, and by using that combined data, they trained a GCN-based system to differentiate among three cancer subtypes, taking into account the known classifications of the cells that are similar to those of the inputs.

In [133], two types of sequential data were combined: one-hot encoded DNA sequences and DNase-Seq signals. The latter one was a real-valued vector that was of length equal to the DNA sequence, showing which regions of the DNA sequence and to what degree that they displayed chromatin accessibility, an epigenomic property implying that these regions played functional and important roles in the specific cell the sample from which it was procured. The task was binary multi-output, with each output representing an epigenetic marker, that took a binary value depending on whether the sample acquired that property. One CNN took the one-hot encoded sequences, while another CNN took the peak signal, and their outputs concatenated and led to dense layers that produced the multi-output classifications. The proposed architecture surpassed the previous state-of-the-art DL models, and the coupling of the genomic sequence with the epigenomic signal resulted in a higher performance than any of the single omics techniques did alone.

In [134], knowledge discovery in cardiovascular disease data was pursued by the unsupervised modeling of multi-omics data. Mice received induced cardiac hypertrophy, while their protein and metabolite levels were monitored over time and recorded to form a dataset. The data of the healthy control subjects were also collected. The goal of the project was to identify the differences between the healthy and cardiac hypertrophy subjects, and to elucidate the pathways and interaction networks of the proteins and metabolites that play a role in cardiovascular disease. Two unsupervised approaches were utilized to extract the patterns out of the data. First, an LSTM-based variational AE extracted the low-dimensional embeddings of the sequential data, and this was followed by k-means clustering. Second, a DL-based clustering model, Deep Convolutional Embedded Clustering (DCEC) [135], took the time-series vectors that were represented in the form of line-plot graphs, wherein a variety of line widths and image sizes were tested, and then, the authors performed clustering. Additionally, conventional clustering algorithms were applied to the original data for a comparison to be made. The results were validated through the Reactome knowledgebase [136], which was used to compare the proteins and metabolites that were clustered together with the known pathways and hierarchical relationships contributing to cardiac disease. The research revealed that DL-based clustering yielded biologically meaningful results, and it surpassed all of the other approaches.

Another domain in the computational life sciences that exploits the use of deep neural networks and multi-omics data is drug development. In [137], the cells were treated with various chemical compounds. Gene expression data were taken from the cells, and they were concatenated with one-hot encoded gene ontology annotations and categorical information that was based on the attributes of the genes. A DNN was trained to predict whether a chemical compound affected a gene to a statistically significant level or not, reaching an AUC of up to 0.84. In [138], a model was designed that took omics data from a biological sample plus information on two drugs and predicted whether the two drugs would have a synergistic effect of treating the cancer type that was expressed in the omics data. The gene expressions, the CNVs, and the somatic mutations of various cancer cell lines were coupled with the data on the physicochemical properties of drugs that are known to target the corresponding cancer cell lines. The drug profiles contained both the real-valued and categorical data, and the labels consisted of synergy scores for the pairs of drugs of the corresponding cell lines. Each of the three types of omics data were fed into a separate AE for their feature extraction. The extracted features were concatenated with the features of any two drugs into a DNN that varied depending on the cancer type, and it always better than the previous state-of-the-art methods did.

In [139], the researchers concatenated four types of data, and they trained a DNN to predict the drug–target interactions (DTI), i.e., whether a drug interacted with the target or not. The four used data types were: (1) the Drug-perturbed gene Expression Profiles (DEPs), where the gene expression was measured for the samples that received chemical treatments, as well as for the control, untreated samples, (2) the Gene-knockdown Expression Profiles (GEPs), where the samples had their genes removed, which represent the gene expression profiles that show how the elimination affected the other genes, and the profiles of the control samples were also taken for comparison, (3) the Protein–Protein-Interaction (PPI) networks, which were graph data that were embedded into vectors via Node2Vec [140], and (4) the pathway membership data, which were embedded through GloVe into vectors that associated together the genes that were functionally related, thereby grouping them into biologically relevant clusters. The first type of data referred to the drugs, while the other three to biological systems and the concatenation of these were used by a DNN to model the drug–target interactions.

In [141], a DNN, along with the conventional ML models, utilized multi-omics data to predict the novel targets for a therapeutic treatment in the field of oncology. Starting from the lists of the genes that were either targeted by the FDA-approved drugs or those which, when they are mutated, may cause cancer, the researchers collected the data for the gene expression, the gene mutations (averaged over numerous patient samples for each cancer type), the gene essentiality (real-valued, mean sensitivity from knock-out experiments), and the gene interaction networks that were embedded via AE-based diffusion graphs [142]. The same data were also collected for the genes that were not present in the therapeutic target or suspicious genes list, thus they made up the negative samples of the dataset. The random forest feature selection reduced the dimensionality of the dataset, and the predictive models were trained, which included a neural network with an output neuron that was activated by a softmax activation function. After learning the positive and negative gene distributions and the interaction networks of the associated genes, the model was given data for any gene and, with it having an AUC of 0.88, it revealed the probability of the gene as a potential target for anticancer drugs. In [143], a method was developed for the early prediction of COVID-19 patient survival by combining plasma multi-omics and DL. The precise concentration of 100 proteins and metabolites in the plasma from hospitalized patients was determined, and it appeared distinctively different from that of the control, healthy patients, thus, indicating the difference between the non-surviving patients and the surviving patients. A DL model was developed, which was able to learn from multi-omics regarding the concentration of ten proteins and five metabolites, so as to predict the early survival of COVID-19 patients, thus reporting a 92% accuracy and 0.97 AUC on the hospitalization day.

In [144], the researchers drew on a wide variety of omics data from cancer cell lines and drug data such as proton pump inhibitors (PPIs), differential gene expression, disease-gene association scores, kinase inhibitor profiling, and growth rate inhibition (GR) to construct a graph. They then applied biological knowledge to simplify the graph, i.e., to remove the edges and nodes with an insignificant influence. A GCN took the drug data and predicted the response across a variety of tumors. In [145], a GCN learned a graph of genes that were related to cancer and PPIs, and it was trained with drug chemical structures and multi-omics data of cancer cells, and it learned to predict the drug response, thus it surpassed most of the existing methods. 

The authors in [146] used Generative Adversarial Networks (GANs) and Functional Interaction (FI) networks [147] for the purpose of biologically informed feature extraction. The datasets were comprised of genomics, transcriptomics, and epigenomics data of seven cancer types. Instead of using a regular, fully connected neural network as the generator, the GAN learned a sparser, biologically inspired network that represented the interactions among the features. The latter scheme was shown to obtain more accurate predictions than the existing methods could.

## 4. Challenges

In this section, the biggest challenges in the field are discussed, including the following aspects: the high dimensionality of the data, imbalanced data, the explainability of the models, data shortage and transfer learning, the need for imputation, suboptimal organization and the standardization of the data in the public databases, and the misclassification of it due to mislabeling. The conclusions are also included in the section.

### 4.1. High Dimensionality of Data

A dataset is considered to be high-dimensional when the number of features in it vastly exceeds the number of samples in it. Biological datasets are infamous for their high dimensionality and for being the “small n—large p” type [148]. Gene expression data may have tens of thousands of features, and DNA methylation data may have hundreds of thousands of them. As though individual omics datasets were not highly dimensional enough, multi-omics integration brings an explosive rise in the data complexity. One of the most highly praised advantages of DL, which has contributed to its widespread adoption, is that it does not need feature engineering. Before DL, image classification required handcrafted features, and tabular data were often simplified via statistical tests and traditional ML techniques before they were applied in predictive modeling. Due to its capacity to extract patterns at various levels of abstraction, DL does not need handcrafted features and, in fact, it has often performed better without them, as the complex, non-linear interactions among the features were often filtered out by the previously used techniques. With high-dimensional data, DL loses this advantage. Its high sensitivity to identifying complex patterns, which normally gives DL its edge, leads to overfitting. Hence, feature engineering is back on the table.

Selecting the most significant features is the traditional route, and standard feature selection algorithms have been widely applied to the biological data [149,150,151], as well as domain-specific techniques for transcriptomics [48,49,50,58]. In terms of the statistical tests, the Wilcoxon rank-sum test was preferred over the t-test for its robustness to the outliers [55]. In [56], the median value of each feature for the in-class samples was compared with that of the out-of-class samples, and again, the median was less sensitive to the outliers than the mean was. A Pearson correlation coefficient was used for a regression task by selecting features with the highest correlation with the output variables [152].

Feature extraction with AEs is widespread in predictive modeling in the life sciences, and with multi-omics data, it is almost a standard procedure. In [51], the AEs were compared with other feature extraction and dimensionality reduction methods on biological datasets, and they were shown to perform the best, whereas the other methods lead to information loss. A broad range of AEs have been used in the field: regular AEs [53,125], stacked AEs [152], denoising [124] and stacked denoising AEs [76], factorization AEs [93], and even self-organizing AEs that determine their own structure according to the input data [153]. Sometimes the AE-extracted features serve as training data for another model, and other times the AE itself, after being pretrained to reconstruct the data, has its encoder module converted into a DNN and trained over for the predictive task [112]. The AE-extracted features may also be selected through statistical techniques, such as ANOVA [126] or Cox PH [120,122,123].

Variational Autoencoders (VAE) have been widely applied for omics dimensionality reduction, such as in [154], where the VAE were used to integrate the genomics, transcriptomics, and epigenomics data for the ovarian cancer subtype identification. The VAE was used to extract the features from the combined mulit-omics datasets, and the extracted features, when they were used for the classification, achieved better results than the other dimensionality reduction techniques did. In [155], our different AEs that were used to extract the features from multi-omics data for cancer classification were compared. Among a conventional AE, there was a sparse AE, a denoising AE, and a VAE; the VAE generally performed better than the other AEs did, and it also surpassed the traditional dimensionality reduction techniques such as a PCA and a kernel PCA. In [156], the researchers experimented with different VAE architectures to integrate the gene expression and clinical data for cancer classification. The best results were produced by a hierarchical architecture that combined both of the individual and shared processing of the datasets as they passed through the layers of the VAE. In [134], an LSTM-based VAE extracted the features from time-series data. The researchers wanted to identify, through unsupervised clustering, the proteins and metabolites that are correlated with each other in cardiovascular diseases. Using the data that tracked the protein and metabolite levels over time, which were taken from mice during cardiac remodeling, they found that reducing dimensionality via an LSTM-based VAE helped the k-means clustering method to achieve better results. In [157], the task was to identify the cancer subtypes by the k-means clustering of the gene expression and miRNA expression data. The authors used a Vector Quantized Variational AutoEncoder (VQ-VAE), which quantized the latent space, thus transforming the real-valued input into categorical values. In that way, the non-linear information was captured. As expected, the latter procedure produced data that were more easily distinguishable by the k-means algorithm which identified the cancer subtypes. VAE-based feature extraction has also been used as a noise reduction strategy in the effort to predict the drug response of cancer patients [158]. The responses to the treatment may vary greatly due to the tumor heterogeneity and microenvironment, thus making drug response data extremely noisy. The researchers in [158] found that extracting the features from the gene expression profiles with VAEs resulted in a better generalization of the drug response and a higher performance in predicting the drug response.

At the high level, feature selection and feature extraction are the two methods that are used for omics fingerprinting, i.e., making the data representations be compressed enough to be computationally viable, but also rich and complex enough for each sample to be distinguishable from the others like a fingerprint. Feature selection may reduce the noise in the data and promote its explainability, but it could miss features whose contribution and significance come in non-linear and indirect ways. Feature extraction, on the other hand, may capture all of the complex, non-linear relations in the data, but it may encode some of the noise as well, and any potential for explainability is largely sacrificed as the extracted features are not understandable by humans. Thus, both of these methods have their pros and cons, and scientists should decide based on their particular research project by experimenting with both of the approaches.

When high dimensionality is accompanied by a high volume of data, the computational cost rises substantially. High-performance computing environments are used in both the industry and academia, and Graphical Processing Units (GPUs) are essential in the field of computational life sciences for computer vision. Another option is to use cloud services, especially if the need for heavy-duty resources arises only on occasion. The dimensionality reduction techniques that are discussed above can simplify the data to a nontrivial extent, and render the task more tractable, although the AE-based feature extraction often comes at the price of a reduced level of explainability.

### 4.2. Data Imbalance

A considerable amount of the public data in the biomedical field is inherently imbalanced. Sometimes this may be associated with biology, e.g., a genomic dataset for an enhancer prediction will have an overwhelmingly over-represented negative class as only a few parts of a DNA sequence constitute enhancers [159]. At other times, the class imbalance stems from the data storing process: a cancer database does not contain many non-cancer data, and a drug research database does not have entries for chemical agents that fail to induce a specific effect. Excluding “uninteresting” data from the databases results in a type of publication bias that made a lot of sense before the advent of predictive modeling. Unfortunately, the ML and DL models must now deal with severe class imbalances.

Computational life science researchers have evaluated a number of responses to this challenge, such as using SMOTE [160] to oversample the minority classes [89,161], or training a separate model for each class at the expense of time efficiency [34]. The data augmentation can help in both the small and the imbalanced datasets [162]. Metrics and loss functions for the imbalanced data, in the context of computational biology, have been discussed in [163]. Instead of relying on accuracy as a metric, the area under the precision-recall curve (AUPRC), the Matthews correlation coefficient (MCC), and the F1-score can be used. A weighted, instead of a regular, cross-entropy loss function can be more useful as it penalizes the errors in the minority class.

Researchers have also sought to enrich the minority classes through intuitive methods and “common sense”. In building a model to identify the venomous proteins from amino acid sequences [77], the researchers collected non-venomous samples by querying for the proteins that did not have the word “venom” on their metadata descriptions. In [141], the positive samples were data of genes that were targeted by drugs. For the negative samples, the researchers used a random subset, of the same size as the positive subset, of genes that were not listed as known therapeutic targets for FDA-approved drugs. The resulting dataset almost certainly contained false negatives, but the researchers assumed that the ratio of false negatives-to-true negatives was minuscule.

For certain domains, such as drug-target interaction databases, removing the publication bias could be worth considering. We might start including all of the “boring” data that were previously left out, although this would inflate the storage space requirements and the time complexity of performing a search in the databases. Perhaps separate repositories for the negative samples could bring the best of both worlds by keeping the regular databases manageable, while also supplying the predictive models with the indispensable negative samples.

### 4.3. Explainability

Deep neural networks are known as black boxes. One cannot know how a neural network reaches its decisions; the parameters of its hidden layers are meaningless to the user. In high-stake biomedical decisions, such as a cancer diagnosis, explainability is of utmost importance. A human medical expert can explain their rationale for making a certain diagnosis, but a neural network cannot be probed to reveal its inner workings. It will generate the predictions, but it will provide no basis for them. While explainability is important in biomedical decision-making, in knowledge discovery it is essential. The generic classification and outcome prediction is one task, while the elucidation of the patterns and relationships among the variables that lead to the classification or outcome is another. Knowledge discovery, when it is pursued through DL, becomes almost synonymous with model explainability.

With the biological sequence data, such as DNA and protein strings, visualizing the activations of the first convolutional layer of a CNN [28] or interrogating the attention layer of an RNN [38,96,113] reveals the subsequences that the model uses to differentiate among the classes. The degree to which the attention mechanisms provide real explainability is contested [164], but in any case, identifying the regions of a sequence to which the model assigned the greatest amount of importance, can be helpful, perhaps with an expert evaluating the findings, or with a literature search validating them.

In [12], semi-restricted bimodal DBNs were trained with proteomics data, with binary values signifying whether a protein was phosphorylated under different stimuli. The interactions among the proteins were inferred by the strength of the DBN’s edges. For each protein, the three strongest edges were picked, thus leading to an inferred protein interaction network which, after a literature review, was proved to be accurate.

In [13], the metabolic profiles were classified with a DNN. The primary purpose was to identify the biomarkers. The researchers identified the important variables by running the DNN multiple times, and each time, they chose one variable and permuted the values, thus each sample assumed the value of another sample on that one column. They calculated the loss of accuracy for each variable and identified the variables whose disordering degraded the performance the most.

In [165], a VAE for classifying cancer from the gene expression profiles was modified to identify which genes contributed to the classification. As the VAE learned to reconstruct the data, a classifying neural network that was connected to the bottleneck layer learned the mapping between the input data and the labels. Then, by using the Deep SHAP methodology [166], they determined the contribution of each input feature to the classification, thus, they identified the most important genes for DL-based cancer detection.

### 4.4. Data Shortage and Transfer Learning

Although the scientific community has generated and curated enormous amounts of biological data over the past decade, given the immense complexity and variety of the biological systems and the numerous subfields and tasks in the field of life science, there will be applications for which there are not enough data. Certain data-capturing technologies may be too expensive to produce the number of samples that a DL model requires, and rare cells or tissues may be hard to collect in adequate amounts for them to be analyzed. Bootstrapping, the traditional statistical solution to a data shortage, was used in [108] to train an ensemble DNN with metabolic profiles, with each DNN having taken different sets of the input data, and the predictions were combined for a regression task. In [14], a U-net took the aggregate ATAC-Seq epigenomic signal of 28 rare cells and predicted what the aggregate signal of 600 cells would look like. In [112], an AE learned to reconstruct metabolic profiles, and then the decoder module was substituted with a dense layer, thus, the AE was converted into a DNN and it was trained for classification. The encoder module had already the data distributions encoded into its synaptic weights, and during the DNN training, the researchers only needed to fine-tune these weights, thus enabling it to learn more efficiently.

In [167], the data shortage problem was addressed with GANs. The researchers wanted to apply DL to detect a brain-related disease using the gene expression data, but the number of training instances was too small for adequate training to occur. Their proposed solution was to first train two GANs to learn the distributions of the input data; one GAN was used for the disease data, while the other one was used for the non-disease data. After the GANs had been trained, the proposed application focused to receive a gene expression profile and to output a prediction score, thus representing the similarity between the input vector and the distributions that were learned by the two GANs. The class, whether it was the disease type or the non-disease type, of the GAN with the highest score, was assigned to the input vector.

A common DL-specific approach in dealing with a data shortage is transfer learning. By iteratively modifying their parameters to fit the data, the neural networks capture the information about the input distributions. Then, given that they have similar data and labels, they do not start from scratch, but rather, the fine-tuned parameters are already calibrated to the model patterns with similar distributions and interactions among the variables, thus they learn the new patterns quickly and without requiring as many samples. As it is a promising and highly practical technology, transfer learning is being vigorously researched in the DL community [168,169]. Computational biology and pharmacology seem to be catching on, with researchers exploiting the technique whenever it is applicable [170,171,172]. In [162], the researchers wanted to map the relationship between the drug chemical structure and the drug activity. Although, quantitative structure property/activity relationship (QSPR/QSAR) tasks suffer from a data shortage, and the neural networks do not get the chance to learn good representations. Therefore, the researchers [84] trained an LSTM with one million unlabeled molecules to learn the representations, and then, they retrained it in a supervised manner with the data for the specific QSPR/QSAR task of interest.

Although transfer learning has not yet been widely applied in DL-based biology, it would be viable and useful for the same reasons that drugs may be repurposed, and the experimentation of them on model organisms yields insights that are applicable to humans. Life has a lower degree of variance than it has been commonly acknowledged to have. Despite its vastness, the fitness landscape of the biological systems is tightly constrained, and it is a search space that is characterized by local optima and dead ends [173,174,175,176]. There are many configurations that matter can take to produce sustainable life. For all of their diversity, organisms develop under tight parameters, common denominators, and the data that are captured from different biological systems will often exhibit similar patterns. Transfer learning can exploit that, and its adoption in the field of life sciences bears the promise of overcoming the challenges of there being a data shortage.

### 4.5. The Need for Imputation

The need for imputation may arise when one is integrating data from different databases with missing values of different entries on the combined dataset. For such a case, a team of researchers in [177] imputed the missing data with Shape Boltzmann machines, which learned the overall distributions, then regenerating the data with the blanks having been filled in. In [178], the authors used GANs to impute the missing values from faulty gene expression data. Imputation is also called for when the data of interest are expensive or technically difficult to gather, but it can be inferred from other data that are cheaper or easier to obtain. The data of interest can be seen as missing values and imputing them through the available data is defined as a multi-variate regression problem. In [179], the epigenomics and genomics data, namely DNA methylation and copy number variants, were transformed into latent-space representations via a denoising AE, and then a DNN generated the gene expression profiles. The measurement of protein abundances is an expensive, relatively novel, and immature process, with only a few proteins being measured at a time, which has a high-costs. On the other hand, the proteins serve as therapeutic targets for drugs and they often constitute the data of interest, and the gene expression data are used to predict the protein expression levels [152,180].

### 4.6. Suboptimal Organization and Standardization of Data in the Public Databases

Training a DL model either for a multi-omics classification or for converting one omics type into another will often require the researchers to combine the data from different repositories. These data may come in different formats, contain duplicate entries and missing values, and adhere to non-uniform metadata annotation schemes. They are hard to curate since the volume of the data is immense and new data come in faster than the curators can handle it. This becomes especially problematic with multi-omics integration. Harmonizing the data could require the application of different normalization, scaling, and transformation procedures for each omics type. Matching the database identifiers may also pose problems when the databases use different or outdated IDs or when there are no one-to-one mappings, which is when one maps a gene to the proteins that it codes for. Over the last few years, the awareness of these issues has risen, and the community is currently examining the logistics of integrating data from heterogeneous sources [181]. Allocating more resources for the curation of them, raising the acceptable quality threshold for new data to be published, and standardizing the formats and metadata annotation schemes are some possible steps in that direction.

### 4.7. Misclassification Due to Mislabeling

In highly complex protein–protein interaction networks, gene regulatory networks, metabolic pathways, etc., errors could be found either in the annotations or the entity at hand (protein, chemical agent, etc.) may possess a functionality that has not been annotated or even discovered yet. Deep neural networks, with their uncanny ability to extract patterns from complex data, may infer some of these functionalities, but their predictions will be rejected due to the faulty annotations; this is currently being exploited by DL-based research in drug repurposing, whereas a drug that is being repeatedly misclassified may share characteristics with drugs from the ’wrong’ class, and its application for other purposes may be warranted [58,61]. The researchers from other fields should be mindful of this effect as well. Seemingly, wrong but persistent classifications should not be immediately rejected. They should first be evaluated, perhaps validated with the existing literature and domain knowledge, and if they appear to be interesting, they could be treated as hypotheses for experimental testing. The databases should be revised whenever such findings are publicized, which often entails researchers who made the discovery taking the initiative and contacting the data repository moderators.

## 5. Discussion and Conclusions

### 5.1. Discussion

The deduction that DL will be increasingly applied in biotechnology cannot be avoided. On one hand, research in biology, biomedicine, and biotechnology is of tremendous value to humanity and, given the modern scientific breakthroughs that open up new avenues, this research is expected to take off. On the other hand, the volume and complexity of the data that are generated render DL the only viable method for extracting these insights as we are dealing with levels of complexity whereby traditional machine learning and statistical techniques often break down. This should be taken into account by the community, and the data gathering procedures of the public databases should be more “DL-friendly” when it is possible, e.g., storing “uninteresting” data to alleviate the class imbalance, standardizing the formats, and performing preprocessing or normalization practices, etc. We are entering an era in which the data that cannot be utilized by DL will be less valuable than those that can.

The future prospects of this area of research become clear when we put things in context in the relevant time frame. The human genome was first fully sequenced in the 2000’s [182], sequencing technologies became practical during the 2010’s [183,184], and the Human Proteome Project made its first release in 2020 [185]. For all of the practical purposes, the field is just starting, and major breakthroughs are yet to come. DL only became a subject of serious study and wide application after 2012, when the Imagenet challenge was solved, and DL proved its real-world efficacy [186]. The coupling of DL with the biological data that are generated through modern high-throughput technologies is a novel scientific discipline that is taking its first baby steps.

As evidenced in Figure 1, the number of papers combining DL and biological data has risen substantially over the last three to five years. Numerous major and ambitious research projects that have taken place during the last couple of years have used DL in at least some part of the process. In their late endeavor to explore and elucidate the proteome landscape across all of the kingdoms of life, a cohort of researchers employed Bidirectional LSTMs to infer the attributes of previously unknown proteins [187]. The DL models take up the front lines in the battle to understand, prognosticate, and cure COVID-19 [188,189,190,191]. Precision medicine and stratified healthcare, and the prospect of exploiting big data to provide customized treatments, rely heavily on deep neural networks [192,193,194], and early cancer prognosis via the use of omics data and DL changes the game in oncology [195].

Even with a limited understanding of the biological concepts, computer scientists can contribute to biotechnology and biomedical research. A grasp of the relevant data types that are used is that which they need to bring their unique expertise on the table. The modern technological breakthroughs have caught experimental biologists by surprise—a pleasant surprise, perhaps, but now, the data and discoveries arrive at a fast pace, and the expanding biological knowledge leaves little time for life scientists to keep up with the computational methods that are capable of exploiting the data. DL and predictive modeling are evolving equally quickly, whereby the scientific progress is reaching the point at which specialization becomes inescapable. The introductory reviews and short primers on DL that are aimed at biologists are valuable and much needed, and if the fields of DL and biology were not so immense and fast-evolving, these primers would be enough. However, the goal of sustainable progress requires computer scientists to get involved in it.

As the involvement of DL in big data analysis in the near future is unquestionable, it is essential to make it accessible to a wider range of researchers. Hopefully, this study, which is aimed at familiarizing DL experts with some of the current biological data and concepts, nudges the scientific community to set off a series of publications addressing computer scientists, inviting them to participate in life science research. Alternatively, this study could be also useful to allow the researchers of biology with no background in computational sciences to understand and utilize the power of DL to gain better insights into and extract important information from the omics data that are available in the field.

### 5.2. Conclusions

In this review, the various types of omics data that are used to utilize DL models and solve the problems in life sciences are examined. The way in which data is prepared for model training, which model architectures are used, and which tasks are addressed are also reviewed. DL holds many promises for scientific breakthroughs, but its potential comes with some shortcomings. The challenges in applying DL to the biological data are discussed, along with ways that these challenges are addressed, and this is an area of vigorous, cutting-edge research. Finally, the future prospects of this domain are considered, and the evidence is shared, suggesting that the activity of it will increase. The conclusion of this review is that the field of life science needs non-biologist DL experts to engage with it, and to get started with this, they need to acquaint themselves with the basics of the biological data. Hopefully, this survey contributes towards this end and aims to inspire researchers to undertake similar efforts.

## Figures and Tables

**Table 1:** Information regarding the five omics levels examined in this study.

Omics Level	Summary Description
Genomics	The genetic information of an organism, its DNA
Transcriptomics	Which pieces of genetic information (genes) are being expressed in a cell
Proteomics	The study of proteins
Metabolomics	The chemical byproducts of cell metabolism
Epigenomics	The three-dimensional structure of DNA, and the various modifications applied to it

**Table 2:** Categories of tasks being solved with omics data and deep learning.

	Description	Examples of Tasks
Biological	Modeling for biological insight or for prediction of biological attributes	Identify functional units in DNA sequences Elucidate interactions among genes or proteins Predict attributes of proteins or DNA sequences
Biomedical	Modeling related to understanding disease, diagnostics, and therapeutics	Cluster biological samples into cancer subtypes Predict whether patient is low- or high-risk Identify biomarkers for early-stage disease
Drug Research	Modeling directly assisting the discovery and development of pharmaceutics	Predict the effect a chemical agent will have on cells, gene expression, or proteins From gene expression profiles, predict which chemical agent was applied on tissue
Bioinformatics	Modeling to assist or automate technical bioinformatics tasks	From a stack of contigs generate consensus sequences From a batch of mass spectra generate a single integrated spectrum Separate peaks from noise in spectra data

**Table 3:** Most prevalent omics data types used to train deep learning models.

Omics	Data	Type	Encoding
Genomics	DNA sequences	String	One-hot k-mers Embedding layer
Transcriptomics	Gene expression profiles	Numerical tabular	
Proteomics	Protein sequences	String	One-hot NLP-based encoding (e.g., skipgram) Encodings based on physicochemical properties Position Specific Scoring Matrix (PSSM)
	Mass Spectrometry	1D vector, time-series	-
Metabolomics	Spectra (various types) Metabolic profiles	1D vector, time-series Numerical tabular	-
Epigenomics	Epigenomic signals Epigenomic profiles	1D vector, time-series Numerical tabular	-

**Table 4:** Example of a PSSM matrix.

Amino Acids	1	2	3	4	5	…
A	0.34	−0.06	0.34	−0.06	−0.06	…
G	−0.06	−0.06	−0.06	−0.06	−0.06	…
I	−0.06	0.18	−0.06	0.34	0.18	…
L	−0.06	0.34	−0.06	−0.06	0.34	…
P	−0.06	0.27	0.18	−0.07	−0.06	…
…	…	…	…	…	…	…

**Table 5:** Example metabolic profile table.

Samples	Compound 1	Compound 2	Compound 3	…	Class
Sample 1	0.871	0.330	0.100	…	o
Sample 2	0.000	0.000	0.306	…	x
Sample 3	0.000	0.453	0.185	…	x
…	…	…	…	…	…

**Table 6:** Example of DNA methylation data.

Samples	Site 1	Site 2	Site 3	…	Class
Patient 1	0.847533	0.110056	0.837559	…	o
Patient 2	0.742208	0.600523	0.953700	…	o
Patient 3	0.064494	0.914458	0.825000	…	x
…	…	…	…	…	…

**Table 7:** Example of somatic mutations data.

Samples	Gene 1	Gene 2	Gene 3	…	Class
Patient 1	0	1	0	…	o
Patient 2	0	0	0	…	o
Patient 3	0	1	1	…	x
…	…	…	…	…	…

**Table 8:** Example of Copy Number Variants (CNV) data.

Samples	Gene 1	Gene 2	Gene 3	…	Class
Patient 1	−1	1	−1	…	o
Patient 2	0	0	0	…	o
Patient 3	0	−1	−1	…	x
…	…	…	…	…	…

## Data Availability

Not applicable.
